# The efficacy of three-dimensional conformal radiation therapy on pain and quality of life in patients with painful bone metastases: a prospective study

**DOI:** 10.3325/cmj.2020.61.215

**Published:** 2020-06

**Authors:** Ayat Faris, José Expósito, Antonio Martínez-Única, Juan Pedro Arrebola, Francisco Miguel Pérez-Carrascosa, Rosario Guerrero, Isabel Tovar

**Affiliations:** 1 Department of Radiology and Physical Medicine, University of Granada, Granada, Spain; 2 Radiation Oncology Department, Virgen de las Nieves University Teaching Hospital, Granada, Spain; 3 Instituto de Investigación Biosanitaria, Granada, Spain

## Abstract

**Aim:**

To evaluate the efficacy of radiation therapy in alleviating pain and improving the quality of life (QoL) with validated questionnaires in patients with painful bone metastases (BoM).

**Methods:**

This prospective, observational study recruited 167 patients with painful BoM who were treated with palliative radiotherapy (PRT) from February 2015 to February 2018. After the first clinical assessment, the patients filled out specific questionnaires and underwent a fast radiotherapy treatment within 48 hours. The patients were followed up for eight weeks.

**Results:**

The median age was 66.30 years. The most common primary cancer was lung cancer (31.1%). The most often prescribed scheme was 8 Gy in one fraction (70%). The patients experienced significant pain response and improved QoL compared with baseline, especially in the first two weeks after radiation. Overall, reduced pain and drug score were reported at two weeks of PRT in 68 (51.5%) and 37 (28%) of patients, respectively.

**Conclusions:**

PRT is an effective treatment option for patients with painful BoM.

Bone metastases (BoM) are a common health issue, as the bone is the third most common metastatic site of cancer (1). It is the most common metastatic site for breast cancer, and most women who died from the disease had BoM at death (2). About 83% of patients with BoM complain of pain at some point in their life, with wide variation in the pattern and intensity of pain (3). BoM are a major cause of morbidity, usually leading to pain and a decreased quality of life (QoL) (4). Common consequences of BoM are bone fractures, which can be prevented or treated by radiation or surgery (5). Palliative radiotherapy (PRT) reduces pain in the majority of patients although it often takes effect only after several weeks of treatment (6). The conventional treatment of BoM is local-field radiation therapy, leading to improvement in about 80% of patients (7). Patients with BoM can suffer from emergency conditions, such as spinal cord compression and uncontrollable bleeding. In these cases, RT should start as soon as possible, often on the day of consultation and simulation, as treatment delay may cause unrecoverable functional loss (8). However, painful BoM is considered a non-emergency condition, and due to increased workload in the radiation unit RT may be delayed for weeks. This is why it is necessary to provide a rapid pathway from primary consultation to RT delivery. In our unit, we introduced an organized rapid pathway to deliver PRT within 48 hours after admission to the unit.

In 1991, Bruera et al (9) developed an instrument that facilitated and standardized the assessment of patients with painful BoM, the Edmonton Symptom Assessment Scale (ESAS), which evaluates nine physical and mental symptoms on a Likert scale. Twenty-two years later, Carvajal et al (10) validated the ESAS as a Spanish reference tool for symptom assessment in advanced cancer patients. Palliative care researchers widely used the European Organisation for Research and Treatment of Cancer Quality of Life Questionnaire Core 30 (EORTC QLQ-C30) (11,12), but it has the disadvantage of being too long. This is why an abbreviated 15-item version, the EORTC QLQ-C15-PAL, was created for use among patients with short life expectancy and advanced, symptomatic cancer (13). The abbreviated version was shown as a reliable and valid instrument for Spanish patients (14). Still, there is limited data on the efficacy of radiotherapy for palliating pain from BoM among patients nearing the end of life (15) owing to the lack of follow-up studies in this population. The systematic review by McDonald et al (16) on the use of RT for BoM included 26 articles, however, the included studies were retrospective and almost all of them lacked a prospective follow-up.

The aim of this study is to present our experience in treating and following up patients with painful BoM and to estimate the efficacy of PRT in improving QoL with validated instruments and in alleviating pain response with visual analogue scale (VAS) as well as the pain and drug scores. Finally, we aimed to assess whether a single or fractional radiation dose was better at improving QoL and relieving pain.

## Patients and methods

### Study design and patients

The participants of this prospective, longitudinal, observational study were recruited among all patients with painful BoM who were referred for PRT in the radiotherapy department of Virgen de las Nieves University Hospital (HUVN) in Granada, Spain from February 2015 to February 2018. This hospital is one of the two public hospitals in the city of Granada, serving a population of over 487 000 people. The inclusion criteria were a proven histological diagnosis of a solid tumor, painful BoM of any bone site that was not previously irradiated, and age older than 18 years. The medical treatment of pain was performed in the pain care unit. Mild pain was treated with a non-steroidal anti-inflammatory drug; acetaminophen, ibuprofen, and indomethacin. Moderate to severe pain was treated with opioid therapy; with mild narcotics tramadol and codeine and strong narcotics fentanyl (especially patches), methadone, and morphine.

Patients with BoM were closely monitored for eight weeks, during which QoL and pain were assessed. After a clinical assessment, PRT was evaluated, and a scheme for RT delivery was outlined on a day-care basis. Following this, a pre-treatment computed tomography simulation was done, and clinical target volume and planning target volume were determined according to recommendations (17). The patients underwent three-dimensional conformal radiation treatment, which is a well-recognized palliative treatment for painful BoM (7). The treatment schemes were from 8 Gy in one fraction to 30 Gy in 10 fractions. All patients previously diagnosed with BoM and referred for PRT during the recruitment period were eligible for participation – 169 in total. The primary tumor sites were the lung (31.1%), breast (15%), urinary bladder (10.8%), and other less frequent tumor sites. Out of the 169 patients, two were not included in the final sample: one rejected to participate and the other died before treatment, leaving a final sample size of 167. All the patients provided an informed consent. The study was approved by the Ethics Committee of the Investigation of the Province of Granada (PI-0439-2014).

### Data collection

Quality of life was evaluated with the EORTC-QLQ-C15-PAL, a validated questionnaire for QoL research in cancer patients that consists of 15 questions with answers on a four-point scale. Patients had to choose the response that best described their state, from 1 (not at all) to 4 (very much). Overall QoL was rated from 1 (very poor) to 7 (excellent) (18). We also used the ESAS, an 11-point scale that evaluates nine symptoms on a scale of 0-10 (0 = absence of symptom and 10 = worst possible symptom). The investigated symptoms were pain, fatigue, nausea, depression, anxiety, drowsiness, appetite, sense of well-being, and shortness of breath. This tool was successfully validated in cancer patients (19-21). Pain response was evaluated according to Chow et al as follows: “complete pain response was delimited as a VAS score of 0, partial response was delimited as pain reduction ≥ 2 at the treated site measured with the VAS (0-10) and without analgesic increase, or as analgesic reduction ≥ 25% from baseline without worsening of pain” (22). The pain response was measured with 1) VAS of pain intensity that ranges from 0 to 10 (0 – no pain, 1 to 3 – mild pain, 4 to 6 –moderate pain, and 7 to 10 – intense pain), 2) the pain score (pain severity score × pain frequency score) and drug score (drug severity score × drug frequency score) (23,24). Patients with complete or partial pain relief in VAS and/or the pain score were considered to show pain response. All the patients completed the questionnaires by themselves within 24 hours before receiving PRT (baseline). At weeks 2, 4, 6, and 8 they were contacted by an experienced interviewer and completed the same questionnaires by telephone. This call was also used to check the patient’s connection with other units (palliative care, medical oncology, general practitioner) and in some cases to continue with the next step in care. The rates of death, re-treatment, and hospital admissions were assessed.

### Statistical analysis

Qualitative variables at each cut-off point of the follow-up are expressed as frequency and percentages. The significance of the change of the dependent variables during the follow-up was assessed with the Friedman’s non-parametric test. Survival after diagnosis was estimated with the Kaplan-Meier method. For survival analysis, the start date was considered the date when the individual was considered a palliative patient and the end date was the date of death or the end of the study (February 2018). Statistical analyses were conducted using the SPSS statistical software package, version 24.0 (IBM Corp, Armonk, NY, USA).

## Results

The median age was 66.30 years (range, 24-96). Ninety-nine patients (59.3%) were male. A total of 149 patients underwent out-patient treatment, and the average time from the first assessment to the first RT dose received was 2 days (range, 1-5). Regarding PRT, 120 (71.9%) patients received single fraction therapy (SFRT), while 47 (28.1%) received multiple fraction therapy (MFRT). The most frequently prescribed scheme was 8 Gy in one fraction (70%), followed by 20 Gy in 5 fractions (15.6%). The most common site of radiation was the spine (50.9%) (Table 1).

**Table 1:** Characteristics of patients (N = 167) with painful bone metastases treated with palliative radiotherapy

Age	No. (%)
**Years (median, range)**	66.3 (24-96)
**Sex**	
male	99 (59.3)
female	68 (40.7)
**Primary tumor**	
lung	52 (31.1)
breast	25 (15.0)
urinary bladder	18 (10.8)
upper gastrointestinal tract	13 (7.8)
lower gastro intestinal tract	13 (7.8)
prostate	12 (7.2)
renal and suprarenal	7 (4.2)
others*	27 (16.2)
**Treatment site**	
spine	85 (50.9)
hip bone	34 (20.4)
ribs	17 (10.2)
sacrum	12 (7.2)
femur	6 (3.6)
scapula	6 (3.6)
others^†^	7 (4.2)

*Thyroid, parathyroid, uterus, ovary, nasopharyngeal, spine, melanoma, sarcoma, testicle, and unknown.

†Clavicle, humerus, and sternum.

**Figure 1:**
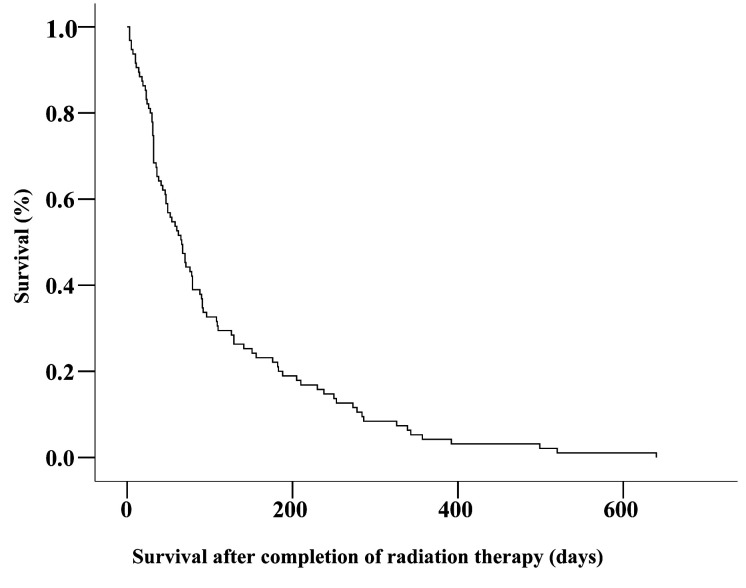
Kaplan-Meier post-radiation therapy survival curve in patients with painful bone metastases. The x-axis shows days until death and the y-axis shows the percent survival.

Sixteen patients were re-irradiated to the same bone site due to pain relapse after an initial satisfactory response. Fourteen patients (8.4%) received a single-fraction dose of 8 Gy, whereas one patient received 20 Gy in 5 fractions and one 15 Gy in 5 fractions (Table 2). Twenty patients (12%) were admitted to the hospital due to severe, unbearable pain during the follow-up period. The post-radiation therapy mean and median survival time was 111 days and 66 days, respectively (95% confidence interval, 86-136 days and 49-82 days, respectively). A total of 37 (22.1%) of participants died within one month of beginning radiotherapy and 157 (94%) patients died by the end of the study (Figure 1). The QLQ-C15-PAL and the ESAS questionnaires were completed before treatment (n = 167) and at week 2 (n = 132), week 4 (n = 103), week 6 (n = 79), and week 8 (n = 68) after radiotherapy. The patients who finished the assessment had significantly improved overall QoL (*P* < 0.001). There was also a significant pain reduction as evaluated by VAS (mean pre-PRT VAS: 7, mean PRT VAS at 2 and 4 weeks respectively after radiation: 5, and mean VAS at 6, 8 weeks respectively: 4; *P* < 0.001) (Table 3).

**Table 2:** Radiation dose in patients (N = 167) with painful bone metastases treated with palliative radiotherapy

Dose (Gy)	Fraction	n (%)
8	1	117 (70)
20	5	26 (15.6)
30	10	6 (3.6)
12	3	5 (3.0)
15	5	2 (1.2)
10	2	2 (1.2)
9	3	2 (1.2)
8	2	2 (1.2)
4	1	2 (1.2)
21	3	2 (1.2)
36	6	1 (0.6)

**Table 3:** Patients’ scores before and after the radiation therapy (RT)*^†^

	Before treatment	At 2 weeks of RT	At 4 weeks of RT	At 6 weeks of RT	At 8 weeks of RT	*p*
**ESAS**						
patients with valid data, n	167	132	103	79	68	<0.001
patients with missing data, n	0	35	64	88	99	
median score (50th)	39	28	25	25	24.50	
**QLQ-C15-PAL**						
patients with valid data, n	167	132	103	79	68	<0.001
patients with missing data, n	0	35	64	88	99	
median score (50th)	31	28	26	26	25	
**VAS**						
patients with valid data, n	167	132	103	79	68	<0.001
patients with missing data, n	0	35	64	88	99	
median score (50th)	7	5	5	4	4	

*ESAS – Edmonton Symptom Assessment Scale; QLQ-C15-PAL – Quality of Life Questionnaire-Core 15-Palliative; VAS – visual analog scale.

†The lower the ESAS and/ or the QLQ-C15-PAL scores, the higher the quality of life. The lower the VAS score, the lower the pain.

**Figure 2:**
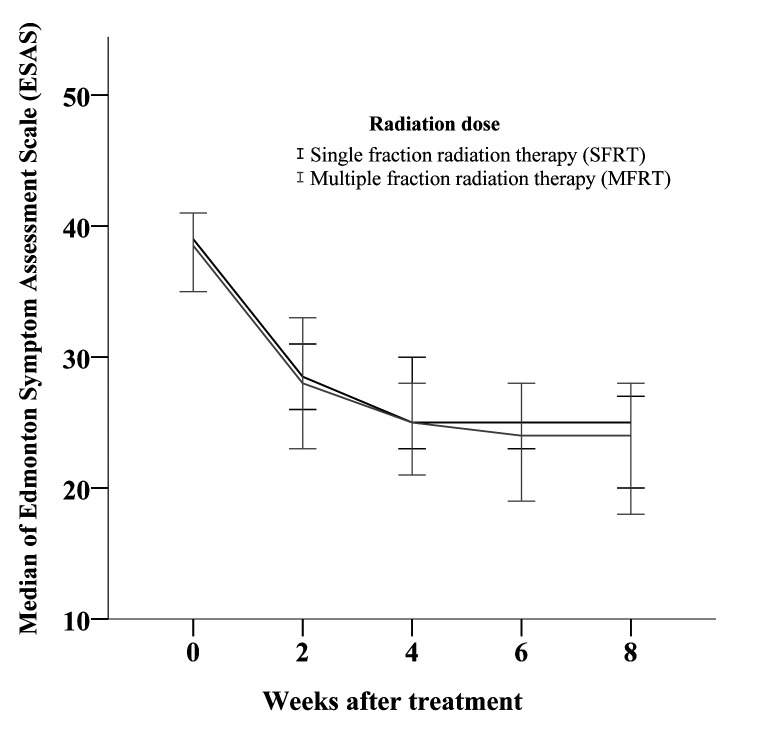
Summarized results of quality of life (QoL) assessment by the median of Edmonton Symptom Assessment Scale (ESAS). The lower the ESAS score, the higher the QoL. The number of patients for single-fraction radiotherapy were 167, 94, 70, 54, 47 and 167, 38, 33, 25, 21 for multifraction radiotherapy. Error bars represent 95% confidence intervals.

Out of 132 patients evaluated at 2 weeks after PRT, 70 (53%) reported partial pain relief as assessed by VAS and 68 (51.5%) as assessed by pain score (Table 4). Moreover, 14 patients (10.6%) had partly reduced analgesic consumption and 23 (17.4%) had discontinued analgesic consumption at 2 weeks (Table 5). The patients who received SFRT did not significantly differ from the patients who received MFRT (Figure 2).

**Table 4:** Pain evaluation in patients (N = 167) with painful bone metastases treated with palliative radiotherapy*^†^

	No. (%) of patients at week			
Pain response after palliative radiation therapy	2	4	6	8

	VAS	pain score	VAS	pain score	VAS	pain score	VAS	pain score
Worse pain	29 (22)	23 (17.4)	19 (18.4)	12 (11.7)	16 (20.3)	7 (8.9)	14 (20.6)	10 (14.7)
No change	33 (25)	41 (31.1)	20 (19.4)	23 (22.3)	13 (16.5)	18 (22.8)	12 (17.6)	12 (17.6)
Partial pain relief	70 (53)	59 (44.7)	63 (61.2)	54 (52.4)	49 (62)	41 (51.9)	41 (60.3)	41 (60.3)
Complete pain relief		9 (6.8)	1 (1)	14 (13.6)	1 (1.3)	13 (16.5)	1 (1.5)	5 (7.4)
valid	132		103		79		68	
missing	35		64		88		99	

*Pain score = pain severity score × pain frequency score. Pain severity (no pain = 0, mild pain = 1, moderate pain = 2, severe pain = 3). Pain frequency (never pain = 0, occasional pain [less than once daily] = 1, intermittent pain [at least once daily] = 2, constant pain = 3). Pain score was defined as complete pain relief = pain score of 0, partial pain relief = pain score decrease ≥ one point, no change = pain score stable before and after treatment, worse pain = pain score increase ≥ one point. Visual analogue scale (VAS) was defined as complete pain relief = VAS score of 0. Partial pain relief = pain reduction ≥2 points at the treated site without analgesic increase, or as analgesic reduction ≥25% from baseline without worsening of pain. No change = VAS was identical when compared with the baseline. Worse pain = VAS increased. VAS and pain score estimate perceived pain in relation to pre-treatment status and, therefore, pre-treatment data were not recorded. Patients with complete or partial pain relief in VAS and/or pain score were considered as showing pain response.

†Significant or borderline-significant differences in VAS score were found between week 2 and week 4 (*P* = 0.042), week 6 (*P* = 0.009), and week 8 (*P* = 0.063). Non-significant differences were found between week 4 and week 6, week 4 and week 8, between week 6 and week 8. Significant or borderline-significant differences in pain score were found between week 2 and week 4 (*P* = 0.005), week 6 (*P* = 0.046), and week 8 (*P* = 0.086), between week 4 and week 6, week 4 and week 8. Non-significant differences were found between week 6 and week 8.

**Table 5:** Drug response in patients (N = 167) with painful bone metastases after receiving palliative radiation therapy (PRT)

	The number (%) of patients with drug score at week after PRT			
Drugs consumption after PRT	2	4	6	8
Increased	12 (9.1)	8 (7.8)	6 (7.6)	4 (5.9)
Stable	83 (62.9)	56 (54.4)	36 (45.6)	33 (48.5)
Partially reduced^‡^	14 (10.6)	14 (13.6)	17 (21.5)	13 (19.1)
Discontinued	23 (17.4)	25 (24.3)	20 (25.3)	18 (26.5)
valid	132	103	79	68
missing	35	64	88	99

*Drug score = drug severity score × drug frequency score. Drug severity: none given = 0, analgesic = 1, mild narcotic = 2, strong narcotic = 3. Drug frequency: none given = 0, less than once per day = 1, once per day = 2, twice or more per day = 3. Drug score was defined as discontinued = drug score of 0, partially reduced = decrease drug score ≥ one point, stable = same drug score before and after treatment, increased = increase drug score ≥ one point. Drug score estimates drug consumption in relation to pre-treatment status and, therefore, pre-treatment data were not recorded.

†Significant differences in drug score were found between week 2 and week 4 (*P* = 0.013), week 6 (*P* = 0.011), and week 8 (*P* = 0.012). Non-significant differences were found between week 4 and week 6, between week 4 and week 8, between week 6 and week-

‡Partially reduced analgesic consumption means that strong narcotic was replaced by a mild narcotic or an analgesic, or that mild narcotic was replaced by analgesic, and/or that the frequency of the drug consumption was changed from twice or more per day to once or less per day.

## Discussion

Our study found pain mitigation and QoL improvement after RT in patients with metastatic bone disease, especially during the first month after radiation. There was no difference between patients who received SFRT and those who received MFRT. This study is the study with the largest number of patients with BoM (167 patients) that used validated QoL instruments (EORTC-QLQ-C15-PAL, ESAS), as well as VAS and pain and drug scores.

BoM is a common problem in patients with tumors and a major cause of disability. Our study presents clinical and evaluative research focused on patients’ outcomes. After one month, 77% of the patients were still alive. After two months, however, only 52% of the patients were still alive and participating in the study. After three years of the study, most of the patients died. The main reasons for not completing the follow-up questionnaires were drastic health deterioration or death. Data were missing for a considerable proportion of patients, most commonly the patients who left the follow-up in the middle of the study.

Most of studies have shown that PRT provides pain relief for about 60% of patients in a median of two to three weeks (25,26). We observed 68% of patients with pain reduction two weeks after irradiation. We also found that the QoL and pain initially improved after treatment and remained stable until the end of the follow-up. This stabilization is an important finding, which reflects the treatment's benefits since untreated patients experience decreased QoL and increased pain. In a retrospective study by Macchia et al (27) involving 160 patients with painful BoM treated with 3D-conformal half-body irradiation (HBI), pain and drug scores were reduced in 76.3% and 50.4% of patients, respectively. In our study, overall pain and drug scores after one month of PRT were 66% and 37.9%, respectively. Although Macchia et al obtained a high percentage of complete responses, the irradiation scheme was longer than the one most frequently used in our study (single dose). The longer scheme is not always feasible in fragile palliative cancer patients who live far from the irradiation center, and we believe treatment schemes should be adapted to patients' clinical condition and life circumstances. Furthermore, in the study by van der Velden et al 61% patients achieved a complete or partial response (28), making their results very similar to ours.

Patients with BoM need a rapid pathway that provides prompt RT delivery in the most comfortable setting. In a previous study conducted in our department, the mean delay from RT unit consultation to RT initiation for BoM was 11 days (29). The mean delay in this study was only two days. This rapid treatment service satisfies the current patients and encourages other patients to choose this method of treatment.

According to Chang et al (30), a radiation oncology model integrating palliative care practice was associated with cost savings, shorter treatment courses, fewer hospitalizations, and increased palliative care. Moreover, Konski et al (31) showed that SFRT was the most cost-effective palliative treatment for patients with BoM from prostate cancer compared with pain medication, chemotherapy, and MFRT. The Andalusian Health Service reported that the treatment cost of RT of a single location BoM with a linear accelerator, and other palliative treatments, up to a maximum dose of 30 Gy was € 1223.67. The cost of the analgesic treatment varies according to patients’ needs, ie, the cost of an implant of subcutaneous morphine infusion pump is € 139.39 (32). Furthermore, Chang et al and Tsang et al showed that shorter RT regimens were most cost-effective in general (30,33). Other studies also showed that SFRT was cost-effective and should be widely adopted for uncomplicated BoM (7). Our study confirms that there was no significant difference in the patient outcome following SFRT or MFRT regimens, although costs alone should not guide treatment decisions and should, therefore, be considered when no significant difference in treatment efficacy is shown.

Palliative patients are specific patients that need all the care that we can offer. They need to be free from pain and distressing symptoms and to feel supported by their health care team. Although patients with BoM generally begin the treatment early enough, a rapid pathway not only accelerates the treatment delivery and enhances the quality of medical service, but also encourages patients to opt for this treatment method.

The study has some limitations, mainly the small number of patients. However, all patients were assessed on a similar basis with validated instruments. In addition, data were gathered from patients with clinical records and treatment sheets, increasing the data reliability.

Our study also introduced the after-treatment use of validated questionnaires: EORTC-QLQ-C15-PAL, ESAS, VAS, and pain and drug scores. In Spain, RT as a treatment option for painful BoM has been applied for years, but there has been a lack of follow-up protocols that use validated QoL questionnaires. This study confirms the effectiveness of the protocol we have used in our center. This model not only improves the QoL and reduces pain, but also makes the work more complete, faster, smoother, and more satisfactory for the patients and working team. Therefore, we believe that our present findings can facilitate the introduction of similar models in other hospitals.

## References

[1] Coleman RE (2001). Metastatic bone disease: clinical features, pathophysiology and treatment strategies.. Cancer Treat Rev.

[2] Solomayer EF, Diel IJ, Meyberg GC, Gollan C, Bastert G (2000). Metastatic breast cancer: Clinical course, prognosis and therapy related to the first site of metastasis.. Breast Cancer Res Treat.

[3] Laird BJA, Walley J, Murray GD, Clausen E, Colvin LA, Fallon MT (2011). Characterization of cancer-induced bone pain: an exploratory study.. Support Care Cancer.

[4] Coleman RE, Rubens RD (1987). The clinical course of bone metastases from breast cancer.. Br J Cancer.

[5] Coleman RE (1997). Skeletal complications of malignancy.. Cancer.

[6] Guadagnolo BA, Liao KP, Elting L, Giordano S, Buchholz TA, Shih YCT (2013). Use of radiation therapy in the last 30 days of life among a large population-based cohort of elderly patients in the United States.. J Clin Oncol.

[7] Lutz S, Balboni T, Jones J, Lo S, Petit J, Rich SE (2017). Palliative radiation therapy for bone metastases: Update of an ASTRO Evidence-Based Guideline.. Pract Radiat Oncol.

[8] Lutz S, Chow E, Hoskin P. Radiation oncology in palliative cancer care. 2013. 1-376 p.

[9] Bruera E, Kuehn N, Miller MJ, Selmser PMK (1991). The Edmonton Symptom Assessment System (ESAS): A simple method for the assessment of palliative care patients.. J Palliat Care.

[10] Carvajal A, Hribernik N, Duarte E, Sanz-Rubiales A, Centeno C (2013). The Spanish version of the Edmonton Symptom Assessment System-Revised (ESAS-r): First psychometric analysis involving patients with advanced cancer.. J Pain Symptom Manage.

[11] Strömgren AS, Goldschmidt D, Groenvold M, Petersen MA, Jensen PT, Pedersen L (2002). Self-assessment in cancer patients referred to palliative care: A study of feasibility and symptom epidemiology.. Cancer.

[12] Kaasa S, Loge JH (2002). Quality-of-life assessment in palliative care.. Lancet Oncol.

[13] Groenvold M, Petersen MA, Bottomley A, Blazeby JM, de Graeff A, Arraras JI (2006). The development of the EORTC QLQ-C15-PAL: a shortened questionnaire for cancer patients in palliative care.. Eur J Cancer.

[14] Arraras JI, Barrondo M, Errasti M, Rico M, de la Vega FA, Zarandona U (2014). The EORTC QLQ-C15-PAL questionnaire: validation study for Spanish bone metastases patients.. Qual Life Res.

[15] Meeuse JJ, Van Der Linden YM, Van Tienhoven G, Gans ROB, Leer JWH, Reyners AKL (2010). Efficacy of radiotherapy for painful bone metastases during the last 12 weeks of life: Results from the dutch bone metastasis study.. Cancer.

[16] McDonald R, Lam H, Soliman H, Chow E, Rowbottom L (2014). International patterns of practice in radiotherapy for bone metastases: A review of the literature.. J Bone Oncol.

[17] Menzel HG (2010). The international commission on radiation units and measurements.. J ICRU.

[18] Caissie A,,  Zeng L, Nguyen J, Zhang L, Jon F, Dennis K (2011). Assessment of health-related quality of life with the European Organization for Research and Treatment of Cancer QLQ-C15-PAL after palliative radiotherapy of bone metastases.. Clin Oncol (R Coll Radiol).

[19] Fan G, Hadi S, Chow E (2007). Symptom clusters in patients with advanced-stage cancer referred for palliative radiation therapy in an outpatient setting.. Support Cancer Ther.

[20] Chang VT, Hwang SS, Feuerman M (2000). Validation of the Edmonton Symptom Assessment Scale.. Cancer.

[21] Moro C, Brunelli C, Miccinesi G, Fallai M, Morino P, Piazza M (2006). Edmonton symptom assessment scale: Italian validation in two palliative care settings.. Support Care Cancer.

[22] Chow E, Hoskin P, Mitera G, Zeng L, Lutz S, Roos DH (2012). Update of the international consensus on palliative radiotherapy endpoints for future clinical trials in bone metastases.. Int J Radiat Oncol Biol Phys.

[23] Caravatta L, Padula G, Macchia G, Morganti G, Ferrandina G, Bonomo P (2012). Short-course accelerated radiotherapy in palliative treatment of advanced pelvic malignancies: A Phase I Study.. Int J Radiat Oncol..

[24] Salazar OM, Sandhu T, Da Motta NW, Escutia MA, Lanzón-Gonzales E, Mouelle-Sone A (2001). Fractionated half-body irradiation(HBI) for the rapid palliation of widespread, symptomatic, metastatic bone disease: a randomized Phase III trial of the international Atomic Energy Agency (IAEA).. Int J Radiat Oncol Biol Phys.

[25] Rich SE, Chow R, Raman S, Liang Zeng K, Lutz S, Lam H (2018). Update of the systematic review of palliative radiation therapy fractionation for bone metastases.. Radiother Oncol.

[26] Sze WM, Shelley MD, Held I, Wilt TJ, Mason MD (2003). Palliation of metastatic bone pain: single fraction versus multifraction radiotherapy–a systematic review of randomised trials.. Clin Oncol (R Coll Radiol).

[27] Macchia G, Ferro M, Cilla S, Buwenge M, Ianiro A, Boccardi M (2018). Efficacy and safety of 3D-conformal half body irradiation in patients with multiple bone metastases.. Clin Exp Metastasis.

[28] van der Velden JM, van der Linden YM, Versteeg AL, Verlaan J-J, Sophie Gerlich A, Pielkenrood BJ (2018). Evaluation of effectiveness of palliative radiotherapy for bone metastases: a prospective cohort study.. J Radiat Oncol.

[29] Expósito J, Jaén J, Alonso E, Tovar I (2012). Use of palliative radiotherapy in brain and bone metastases (VARA II study).. Radiat Oncol.

[30] Chang S, May P, Goldstein NE, Wisnivesky J, Ricks D, Fuld D (2018). A palliative radiation oncology consult service reduces total costs during hospitalization.. J Pain Symptom Manage.

[31] Konski A (2004). Radiotherapy is a cost-effective palliative treatment for patients with bone metastasis from prostate cancer.. Int J Radiat Oncol Biol Phys.

[32] Servicio Andaluz de SaludPrecios Públicos. Available from: https://www.sspa.juntadeandalucia.es/servicioandaluzdesalud/profesionales/recursos-para-profesionales/precios-publicos. Accessed. May 6, 2020.

[33] Tsang DS, Yau V, Raziee H, Niglas M, Soliman H, Chow E (2015). Debate: single-fraction treatment should be standard in the retreatment of uncomplicated bone metastases.. Ann Palliat Med.

